# JNK signaling maintains the mesenchymal properties of multi-drug resistant human epidermoid carcinoma KB cells through snail and twist1

**DOI:** 10.1186/1471-2407-13-180

**Published:** 2013-04-04

**Authors:** Xia Zhan, Xiaobing Feng, Ying Kong, Yi Chen, Wenfu Tan

**Affiliations:** 1 Department of Pharmacology, School of Pharmacy, Fudan University, 826 Zhangheng Road, Shanghai, 201203, P.R. China; 2 State Key Laboratory of Drug Research, Shanghai Institute of Materia Medica, Chinese Academy of Sciences, 555 Zu Chong Zhi Road, Shanghai, 201203, P.R. China; 3 Medical School of Nantong University, Nantong, 226001, P.R. China; 4 Department of Integration of Traditional Chinese and Western Medicine, Peking University School of Oncology, Beijing Cancer Hospital, Beijing, China

**Keywords:** c-Jun N-terminal kinases, Snail, Twist1, Epithelial mesenchymal transition

## Abstract

**Background and methods:**

In addition to possess cross drug resistance characteristic, emerging evidences have shown that multiple-drug resistance (MDR) cancer cells exhibit aberrant metastatic capacity when compared to parental cells. In this study, we explored the contribution of c-Jun N-terminal kinases (JNK) signaling to the mesenchymal phenotypes and the aberrant motile capacity of MDR cells utilizing a well characterized MDR cell line KB/VCR, which is established from KB human epidermoid carcinoma cells by vincristine (VCR), and its parental cell line KB.

**Results:**

Taking advantage of experimental strategies including pharmacological tool and gene knockdown, we showed here that interference with JNK signaling pathway by targeting JNK1/2 or c-Jun reversed the mesenchymal properties of KB/VCR cells to epithelial phenotypes and suppressed the motile capacity of KB/VCR cells, such as migration and invasion. These observations support a critical role of JNK signaling in maintaining the mesenchymal properties of KB/VCR cells. Furthermore, we observed that JNK signaling may control the expression of both snail and twist1 in KB/VCR cells, indicating that both snail and twist1 are involved in controlling the mesenchymal characteristics of KB/VCR cells by JNK signaling.

**Conclusion:**

JNK signaling is required for maintaining the mesenchymal phenotype of KB/VCR cells; and JNK signaling may maintain the mesenchymal characteristics of KB/VCR cells potentially through snail and twist1.

## Background

One of major obstacles for successful tumor chemotherapy is the development of acquired drug resistance, which possesses a property of cross drug resistance, namely multiple-drug resistance (MDR). Many efforts have been made to elucidate the mechanisms of MDR and to develop strategies for overcoming MDR aroused during chemotherapy [1,2]. On the other hand, studies demonstrate that cancer cells survived chemotherapy acquire aberrant metastatic capacity, similar to the phenomena that cancer cells acquire MDR property after exposed to chemotherapeutic drugs [3-5]. In this regard, elucidating the molecular mechanisms underlying aberrant metastatic capacity of MDR cells is quite important, as it may provide new targets for improving the efficiency of chemotherapy.

For metastasis from a primary tumor site, cancer cells must lose cell-cell adhesion and acquire motility to invade adjacent cell layers [6]. This process shares many similarities with epithelial-mesenchymal transition (EMT), which has been proposed as one of critical mechanisms for the acquisition of metastatic capacity by epithelial cancer cells [7]. EMT, a highly conserved cellular program in many important phases of embryonic development, is a biological process through which epithelial cells lose their epithelial cobblestone phenotype and acquire fibroblastic and mesenchymal-like phenotypes. These dramatic phenotypic changes involve disruption of intercellular junctions, replacement of apical-basal polarity with front to back polarity, and acquisition of more potent motile ability [8]. The hallmarks of EMT are characterized by loss of epithelial adhesion molecule E-cadherin and gain of mesenchymal markers such as N-cadherin, vimentin, *et al*. [9]. A wide range of extracellular signaling pathways have been shown to trigger the process of EMT, such as signalings elicited by transforming growth factor-β (TGF-β), fibroblast growth factor (FGF), epidermal growth factor (EGF), *etc*. The stimulation of these signaling pathways results in the activation of numerous transcriptional factors, including snail1 (hereafter snail), snail2/slug, twist1, ZEB1, ZEB2, hypoxia inducing factors (HIF), NF-κB, stat3, stat5 and Foxo2, thereby controlling the alterations in gene-expression patterns that underlie EMT [8,10].

The c-Jun NH2-terminus kinases (JNKs), also called the stress-activated protein kinase (SAPK), are serine/threonine kinases that belong to the mitogen-activated protein kinase (MAPK) family. JNKs are encoded by three genes, *JNK1*, *JNK2* and *JNK3*. Two of these genes, JNK1 and JNK2, are expressed ubiquitously, while JNK3 is selectively expressed in neurons. JNKs can be activated by environmental stresses, mitogens, and oncogenes, and play a critical role in tumor development [11,12]. JNK signaling plays crucial roles in numerous biological processes such as proliferation, differentiation, survival and migration through its downstream effector activating protein1 (AP1), such as c-Jun, JunB, JunD [11]. Much attention has been focused on the contribution of JNK signaling in MDR aroused during chemotherapy [13,14], whereas the contribution of JNK signaling to the aberrant motile capacity of MDR cells and its underlying mechanisms remain poorly understood. This study explored the influences of JNK signaling on EMT of MDR cells to dissect the potential mechanisms underlying the aberrant motile capacity of MDR cells using a well characterized MDR cell line KB/VCR, a subline established from a human epidermoid carcinoma cell line KB by vincristine (VCR), and its parental KB cells [15].

## Methods

### Materials

Rabbit monoclonal phospho-JNK1/2 and c-Jun were purchased from Cell Signaling Technology (Beverly, MA). Rabbit monoclonal antiserum against JNK1/2, c-Jun, E-cadherin, N-cadherin, vimentin, snail, twist1, GAPDH, and β-actin were obtained from Santa Cruz Biotechnology (Santa Cruz, CA); SP600125 was purchased from Sigma (St. Louis, MO). JNK1/2 shRNA (5^′^-CCGGAAAGAATGTCCTACCTTCTTTCTCGAGAAAGAAGGTAGGACATTCTTTTTTTTG-3^′^) and shRNA control cloned into pMAGIC 7.1 lentiviral system were purchased from Sunbio (Shanghai, China). The c-Jun siRNA (5^′^-GCGGGAGGCAUCUUAAUUATT-3^′^) and control were obtained from GenePharma (Shanghai, China)

### Cell lines and transfection

KB human epidermoid carcinoma cells and human epithelial kidney 293 T cells were obtained from the American Type Culture Collection (Manassas, VA) and maintained in Dulbecco’s modified Eagle’s medium (Sigma; St. Louis, MO) containing 10% fetal calf serum. The VCR-selected multiple-drug tolerant KB/VCR subline was obtained from Zhongshan University of Medical Sciences (Guangzhou, China) and routinely cultured in medium containing VCR (200 ng/ml). KB/VCR cells were cultured in VCR-free medium for at least 3–7 days prior to be used for experiments to avoid drug-associated secondary effects, and were cultured in absence of VCR for no more than 15 days to keep the MDR phenotype. The KB/VCR resistant cells were authenticated by comparing their fold resistance with that of the parental cells and examining the expression level of ABC transporters (ABCB1 and ABCG2) in KB/VCR cells cultured in the presence, or absence of VCR for about 15 days.

The c-Jun siRNA and control were transfected to KB/VCR cells using lipefectamine 2000 (Invitrogen; Carlsbad, CA) as the instructions provided by the manufacturer.

### shRNA and lentivirus infections

The JNK1/2 shRNA and control were transfected to 293 T cells using lipefectamine 2000 (Invitrogen; Carlsbad, CA) as the instructions provided by the manufacturer. Viral stocks were prepared and infections performed as previously reported [16].

### Migration and invasion assay

Migration assay and invasion assay were determined using a transwell system (Corning Costar; Acton, MA) with an 8-μm pore size membrane coated with fibronectin for migration assay or with matrigel for invasion assay as described by Li et al. [17]. Briefly, 100 μl (5 × 10^4^ cells) of KB or KB/VCR cells was added to the upper wells and 600 μl of DMEM with or without 10% FBS, which was used as a chemoattractant, was added to the lower wells. After incubation for 8 h at 37°C, the migrated cells or invaded cells were fixed with 90% EtOH and then stained with 0.1% crystal violet in 0.1 mol/L borate and 2% EtOH (pH 9.0). The stained cells were subsequently extracted with 10% acetic acid. The absorbance values were determined at 570 nm with a Spectrophotometer (BioTek; Winooski, VT).

### Reverse transcription-PCR analyses

Total RNA was isolated with the RNAiso Plus Kit from TaKaRa (Dalian, China) as the instructions provided by the manufacturer. Total RNA was reversely transcribed using Moloney Murine Leukemia Virus (M-MLV) reverse transcriptase (TaKaRa; Dalian, China) and cDNAs were used for PCR with the following primers (Invitrogen; Shanghai, China): *Snail*: 5^′^-GAGGCGGTGGCAGACTAG-3^′^, 5^′^-GACACATCGGTCAGACCAG-3^′^; *twist1*: 5^′^-GGAGTCCGCAGTCTTACGAG-3^′^, 5^′^-TCTGGAGGACCTGGTAGAGG-3^′^; *HIF-1*: 5^′^-CAGCTATTTGCGTGTGAGGA-3^′^,5^′^-CCAAGCAGGTCATAGGTGGT-3^′^; *NF-*κ*B*: 5^′^-GGCGAGCAACTCAATAAAGC-3^′^, 5^′^-GAGCAAAGGACTGCCAAGAC-3^′^; *Foxo2*: 5^′^-GATCACCTTGAACGGCATCT-3^′^, 5^′^-ACCTTGACGAAGCACTCGTT-3^′^; *slug*: 5^′^-CTTTTTCTTGCCCTCACTGC-3^′^, 5^′^-ACAGCAGCCAGATTCCTCAT-3^′^; *ZEB1*: 5^′^-GAGAAGCGGAAGAACGTGAC-3^′^, 5^′^-GCTTGACTTTCAGCCCTGTC-3^′^; *ZEB2*: 5^′^-TTCCTGGGCTACGACCATAC-3^′^, 5^′^-GCCTTGAGTGCTCGATAAGG-3^′^; *Stat3*: 5^′^-ACATTCTGGGCACAAACACA-3^′^, 5^′^-CACACCAGGTCCCAAGAGTT-3^′^; *Stat5a*: 5^′^-ACATTTGAGGAGCTGCGACT-3^′^, 5^′^-CCTCCAGAGACACCTGCTTC-3^′^; *TATA*: 5^′^-ACCCTTCACCAATGACTCCTATG-3^′^, 5^′^-TGACTGCAGCAAATCGCTTGG-3^′^. The PCR products were analyzed using agarose gel electrophoresis.

### Real time-PCR analyses

Total RNA was subjected to reverse transcription with the kit from TaKaRa (Dalian, China) according to the manufacturer’s instructions. Then the cDNAs were amplified by Real-time PCR (iQ5; Bio-Rad) with the SYBR-Green kit (TaKaRa, Dalian, China) with the primers (Invitrogene; Shanghai, China) mentioned above. The alteration of mRNA expression in cells was assessed using the iQ5 optical system software by delta delta Ct method.

### Immunoblot analysis

Cells were lysed in lysis buffer (50 mM Tris–HCl, 150 mM NaCl, 1% Nonidet P-40) supplemented with protease inhibitors (0.5 mM phenylmethylsulfonyl fluoride, 1 μg/ml aprotinin and leupeptin) for 15 min on ice. Equal amounts of protein were subjected to SDS-polyacrylamide gel electrophoresis and transferred onto a polyvinylidene difluoride membrane (Immobilon P; Millipore). The membranes were then incubated with the appropriate antibodies as indicated.

### Statistical analysis

The data of the experimental studies were expressed as the average ± s.d. Statistical differences were analyzed by the two-tailed Student’s t test and P < 0.05 was considered as significant.

## Results

### KB/VCR cells exhibit mesenchymal properties and aberrant motile capacity compared to the parental cells KB

**Figure 1:**
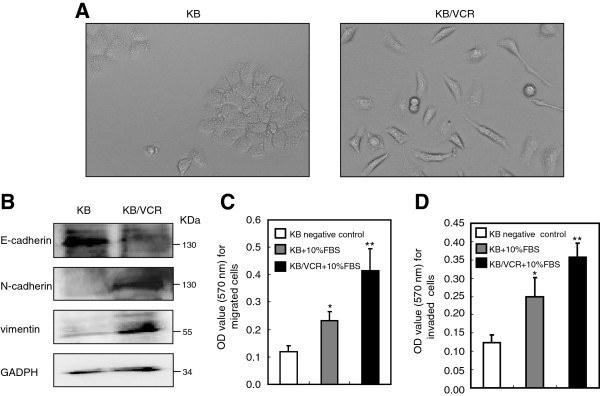
**KB/VCR cells exhibit mesenchymal properties compared to parental KB cells. A.** KB/VCR cells exhibit elongated, fibroblastic morphological alterations, whereas the KB cells displayed epithelial cobblestone phenotype. (×10). **B.** Expression of epithelial markers E-cadherin in KB/VCR cells is decreased, concomitantly with increased expression of mesenchymal markers N-cadherin and vimentin, when compared to KB cells. KB and KB/VCR cells were lysed and used for western blotting analysis, using the GAPDH as a loading control. All blots present in this paper are representatives from at least three independent experiments. Values quantified by Image J from three independent experiments were statistical different.** C-D.** KB/VCR cells possess more potent motile behavior ability, including migration (C: *P < 0.05 compared to KB negative control; ** P < 0.05 compared to KB with 10%FBS) and invasion (D: *P < 0.05 compared to KB negative control; ** P < 0.01 compared to KB with 10%FBS) in response to serum, in comparison to KB cells. KB/VCR cells or KB cells were loaded into the upper wells, while the growth medium containing 10% serum was added into the lower wells of transwell system with a membrane coated by fibronectin or matrigel, and Serum free medium was used as a negative control. After 8 h, the migrated or invaded cells onto membranes were fixed and quantified as described in materials and methods. Values are the average ± SD of results obtained from three separated experiments.

We employed the MDR cell line KB/VCR, a well established MDR cell line [15], to firstly explore whether the MDR cells possess mesenchymal properties. We observed that the KB/VCR cells exhibited elongated, fibroblastic morphology (Figure 1A). On the contrary, the parental cells KB exhibited epithelial cobblestone phenotype (Figure 1A). These observations suggested that the KB/VCR cells lost the epithelial morphology and acquired mesenchymal phenotype when compared to parental KB cells. We next evaluated the expression of markers for epithelial and mesenchymal characteristics in KB/VCR cells and parental KB cells with western blot analysis. In line with the alterations in morphology between KB/VCR and KB cells, we found that the expression of the epithelial marker E-cardherin was decreased in KB/VCR cells compared to KB cells, while the expression of mesenchymal markers including N-cadherin and vimentin was increased when compared to KB cells (Figure 1B). We then sought to compare the motile behavior including migration and invasion abilities between KB cells and KB/VCR cells utilizing a transwell system. Our results revealed that KB/VCR cells migrated and invaded more efficiently than KB cells in response to 10% serum, which was used as a chemoattractant (Figure 1C-D). These data together indicate that KB/VCR cells undergo epithelial-mesenchymal transition and acquire more powerful motile capacity in comparison to KB cells.

### Pharmacological inhibition of JNK1/2 activation with SP600125 reverses the mesenchymal phenotypes of KB/VCR cells

**Figure 2:**
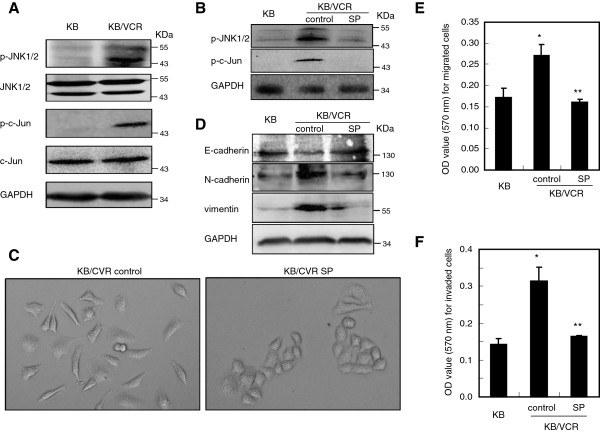
**Pharmacological inhibition of JNK1/2 activation with SP reverses the mesenchymal phenotypes of KB/VCR cells. A.** KB/VCR cells display enhancement level of p-JNK1/2 and p-c-Jun when compared to KB cells. The expression of p-JNK1/2, p-c-Jun and their total proteins was analyzed by Western blot with specific antibodies, using antibody to β-actin as a loading control. **B.** Suppression of JNK signaling by SP inhibits the phosphorylation of JNK1/2 and c-Jun in KB/VCR cells. KB/VCR cells were treated with SP (10 μM) for 24 h, and the proteins were then harvested for western blot analysis as mentioned in materials and methods. **C.** Exposure of KB/VCR cells to SP 10 μM for 24 h renders the KB/VCR cells losing the mesenchymal fibroblastic morphology and recovering epithelial cobblestone phenotype. (×10). **D.** Treatment of KB/VCR cells with SP recovers the expression pattern of epithelial and mesenchymal markers in KB/VCR cells similar to those in KB cells. KB/VCR cells treated with SP (10 μM) for 24 h were lysed and used for western blot analysis of EMT protein markers. Proteins extracted from KB cells were used as a control. **E-F.** Interfering with JNK1/2 by use of SP inhibited the migration (E: *P < 0.05 compared to KB; **P < 0.05 compared to control) and invasion (F: *P < 0.05 compared to KB; **P < 0.001 compared to control) of KB/VCR cells in response to serum. Cells were loaded into upper well, while the SP (10 μM) was added together with medium containing 10% FBS to the lower wells. Data are expressed as average ± SD of results obtained from three separated experiments. In all cases, blots are representative of three to four independent experiments.

JNK signaling has been implicated in the occurrence of MDR during chemotherapy [13,14], whereas there is little knowledge regarding the contribution of JNK signaling to EMT properties and motile ability of MDR cells. We then set out to explore the influence of JNK signaling on the mesenchymal phenotypes of MDR cells. To this end, we firstly assessed whether JNK signaling is activated in KB/VCR cells by evaluating the phosphorylation status of JNK1/2 and c-Jun, a key transcription factor downstream of JNK1/2. Western blot analysis revealed that JNK1/2 and c-Jun were obviously phosphorylated when compared to that in KB cells, whereas there was no alteration in the total protein expression for JNK1/2 and c-Jun. These results suggest that JNK signaling is activated in KB/VCR cells when compared to KB cells (Figure 2A). Keeping this in mind, we then asked whether interference with JNK signaling would disrupt the mesenchymal properties of KB/VCR cells. We began by blockade of the JNK1/2 signaling system through targeting JNK1/2 with a small molecular compound SP600125 (SP), a specific and widely used antagonist of JNKs [18], to inhibit the JNK1/2 activity in KB/VCR cells. As expected, treatment of KB/VCR cells with SP significantly suppressed the phosphorylation of JNK1/2 and c-Jun in KB/VCR cells (Figure 2B), confirming that SP can be useful for investigating the contribution of JNK1/2 in maintaining the mesenchymal phenotype of KB/VCR cells. After treated with SP 10 μM for 24 h, the KB/VCR cells lost the fibroblatic mesenchymal morphology and recovered the epithelial cobblestone phenotype (Figure 2C) similar to that possessed by KB cells as shown in Figure 1A. Meanwhile, exposure of the KB/VCR cells with SP 10 μM for 24 h obviously reduced the expression of mesenchymal markers N-cadherin and vimentin in KB/VCR cells, accompanying with elevated expression of epithelial marker E-cadherin (Figure 2D). Moreover, we observed that the expression of epithelial and mesenchymal markers in KB/VCR cells after treated with SP was almost recovered to that in KB cells (Figure 2D). In line with these alterations in the expression of epithelial and mesenchymal markers, we found that after treatment with SP, the migration (Figure 2E) and invasion (Figure 2F) of KB/VCR cells in response to serum were abundantly suppressed. Taken together, these observations support that JNK1/2 activation is required for maintaining the mesenchymal properties of KB/VCR cells.

### Knockdown of JNK1/2 reverses the mesenchymal phenotype of KB/VCR cells

**Figure 3:**
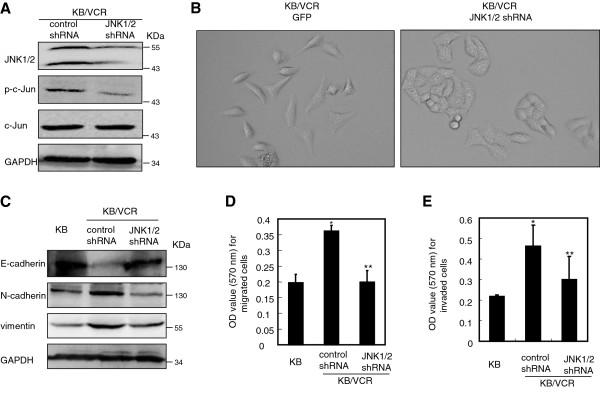
**Knockdown of JNK1/2 reverses the mesenchymal phenotypes of KB/VCR cells. A.** Knockdown of JNK1/2 by lentiviruses carrying JNK1/2 shRNA. KB/VCR infected with lentiviruses carrying shRNA control or JNK1/2 shRNA were lysed after infection, and used for Western blot analysis. Western blot for GAPDH was used as a loading control. Data are similar results from three independent experiments. **B.** Knockdown the expression of JNK1/2 by JNK1/2 shRNA results in KB/VCR cells acquiring of epithelial cobblestone like morphology contrast to that of shRNA control. (×10). **C.** Knockdown of the expression of JNK1/2 significantly increases the expression of E-cadherin and obviously decreases the expression of N-cadherin, vimentin in KB/VCR cells. The expression of E-cadherin, N-cadherin and vimentin was examined as described above in KB/VCR cells after infection with lentiviruses carrying shRNA control or JNK1/2 shRNA. Data are representative blots from three independent experiments. **D-E.** Knockdown of the expression of JNK1/2 significantly inhibits the migration (C: *P < 0.001 compared to KB; **P < 0.01 compared to control shRNA) and invasion (D: *P < 0.05 compared to KB; **P < 0.05 compared to control shRNA) of KB/VCR cells in response to serum. The migration and invasion of KB/VCR cells in response to serum were examined as described above after infection with lentiviruses carrying shRNA control or JNK1/2 shRNA. Data are expressed as average ± SD of results obtained from three separated experiments.

To further address the direct role of JNK1/2 activation in maintaining the mesenchymal characteristics of KB/VCR cells, we set out to knockdown JNK1/2 using RNA interference approach. We designed a shRNA sequence targeting both JNK1 and JNK2 and expressed it stably in KB/VCR cells using a lentiviral system. The western blot analysis revealed that the expression of JNK1/2 in KB/VCR cells infected with lentiviral mediated JNK1/2 shRNA was significantly decreased when compared to that of cells infected with the shRNA control (Figure 3A). Consequently, we observed that the phosphorylation of c-Jun was also reduced as anticipated (Figure 3A). Meanwhile, we found that limiting expression of JNK1/2 resulted in KB/VCR cells re-gaining an epithelial cobblestone phenotype (Figure 3B), which was similar to that of KB cells (Figure 1A) and contrast to the elongated, fibroblastic morphology of KB/VCR cells infected with shRNA control (Figure 3B). In alignment with the results obtained by the pharmacological tool SP, limiting the expression of JNK1/2 led to increase the expression of epithelial marker E-cadherin and to decrease the expression of mesenchymal markers N-cadherin, vimentin, when compared to that of the KB/VCR cells infected with shRNA control (Figure 3C). Furthermore, we observed that knockdown the expression of JNK1/2 obviously inhibited the migration and invasion of KB/VCR cells responding to serum (Figure 3D, E). These observations together with those obtained by small molecular antagonist SP demonstrate that JNK1/2 activation is quite important for maintaining the mesenchymal phenotype of KB/VCR cells.

### Knockdown of c-Jun disrupts the mesenchymal phenotype of KB/VCR cells

**Figure 4:**
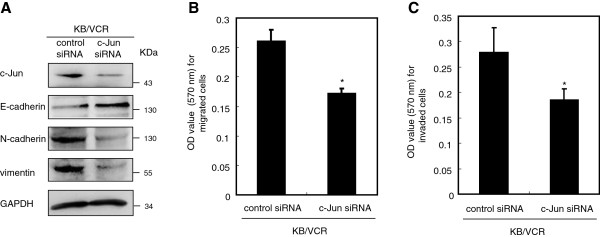
**Knockdown of c-Jun disrupts the mesenchymal phenotypes of KB/VCR cells. A.** Expression of c-Jun, E-cadherin, N-cadherin and vimentin after knockdown of c-Jun with RNA interfering approach. KB/VCR cells transfection of siRNA targeting c-Jun or siRNA control were lysed and used for western blot analysis with specific antibodies. Blots are representative of three independent experiments. **B-C.** Limiting the expression of c-Jun with siRNA reduces the migration (B: * P < 0.01) and invasion (C: *P < 0.05) of KB/VCR cells. The migration and invasion of KB/VCR cells in response to serum were examined as described above after transfection with c-Jun siRNA and the siRNA control. Data are expressed as average ± SD of results obtained from three separated experiments.

The oncogenic function of JNKs is mostly based on their ability to phosphorylate c-Jun [11]. We then further investigated the efficiency of c-Jun on the mesenchymal phenotype of KB/VCR cells by eliminating the expression of c-Jun with small RNA interference approach. As expected, limiting the expression of c-Jun (Figure 4A) caused elevation of the expressions of epithelial marker E-cadherin and concomitantly reduction in the expression of mesenchymal markers N-cadherin and vimentin in KB/VCR cells (Figure 4A), paralleling to the observations achieved via interference with JNK1/2 by pharmacological and shRNA approaches. Consistent with the changes in the expression of epithelial and mesenchymal markers, knocking down the expression of c-Jun also resulted in preventing the migration and invasion of KB/VCR cells in response to serum (Figure 4B, C). These data together indicate that c-Jun, as well as JNK1/2, may be involved in maintaining the mesenchymal phenotype of KB/VCR cells, further supporting the argument that the JNK signaling is strictly required for maintaining the mesenchymal properties of KB/VCR cells.

### Snail and twist1 are both involved in maintaining the mesenchymal properties of KB/VCR cells by JNK signaling

**Figure 5:**
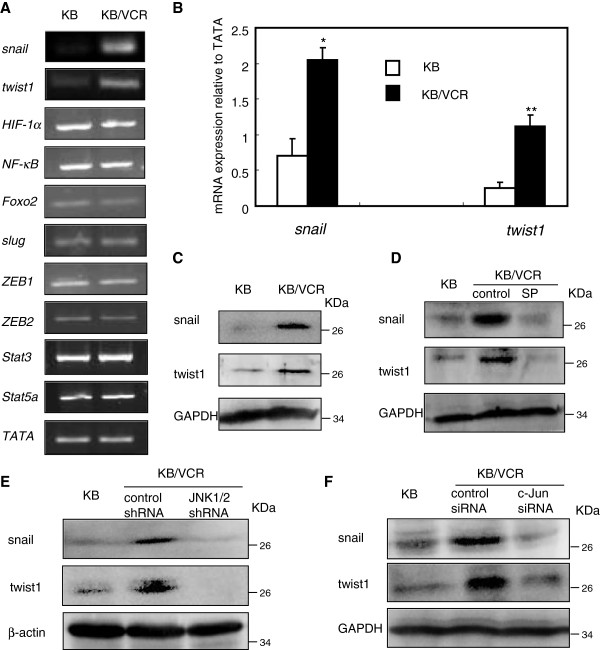
**Snail and twist1 are both involved in maintaining the mesenchymal properties of KB/VCR cells controlled by JNK signaling. A.** RT-PCR analysis for the expression of transcriptional factors associated with EMT in KB and KB/VCR cells. Total RNA of KB and KB/VCR cells was extracted and reversed to cDNA, which was subsequently subjected to PCR analysis with specific primers as indicated in materials and methods. **B.** Expression of *snail* and *twist1* at mRNA level was determined by real time PCR analysis (*P < 0.01; **P < 0.05). **C.** Expression of snail and twist1 at protein level was determined by western blot analysis. **D-E.** Interfering with JNK1/2 with SP (D) or JNK1/2 shRNA (E) suppressed the expression of snail and twist1 in KB/VCR cells. KB cells or KB/VCR cells treated with SP 10 μM after 24 h or after infected with JNK1/2 shRNA were lysed and subjected to western blot analysis using specific antibodies. Western blot for β-actin was used as a loading control. **F.** Knockdown of c-Jun with siRNA reduces the expression of snail and twist1 in KB/VCR cells. KB cells or KB/VCR cells transfected with c-Jun siRNA or control were lysed, and used for western blot analysis with specific antibodies. In all cases, blots are representative of three to four separate experiments.

A wide range of transcriptional factors activated by extracellular signaling have been directly or indirectly implicated in controlling the expression of epithelial and mesenchymal markers and eventually the phenotypes of EMT [10]. We thus sought to explore the potential transcriptional factors involved in the mesenchymal phenotypes maintained by JNK signaling in KB/VCR cells. Using RT-PCR approach, we firstly detected the expression of a panel of transcriptional factors including snail, slug, twist1, HIF1α, NF-κB, Foxo2, ZEB1, ZEB2, stat3 and stat5a, which all have been well characterized in inducing of EMT [10], at transcript level in KB/VCR cells compared to parental ones. Of interest, we observed that there were no alterations in the expression at transcript levels of the majority of those transcriptional factors, with exemption of snail and twist1, whose expression at mRNA level was obviously elevated in KB/VCR cells compared to that of parental KB cells (Figure 5A). This observation was further validated by real time PCR and western blot analysis at mRNA and protein levels, respectively (Figure 5B-C), thus suggesting the possibility of those two transcriptional factors being involved in mediating the EMT controlled by JNK signaling in KB/VCR cells. We then set out to gain direct evidences regarding the involvement of snail and twist1 in the mesenchymal properties controlled by JNK signaling in KB/VCR cells. Blockade of JNK1/2 activation with pharmacological tool SP (Figure 5D) or JNK1/2 shRNA (Figure 5E) approaches caused an obviously reduction in the expression of snail and twist1 when compared to that of control KB/VCR cells. Similar results were observed by mean of silencing the expression of c-Jun with siRNA (Figure 5F). Collectively, these observations suggest that JNK signaling may control the mesenchymal properties of KB/VCR cells through acting on both snail and twist1.

## Discussion

In the present study, we utilized MDR cells KB/VCR [15], a well established MDR cell line possessing high resistance index and its parental one to examine the potential contribution of JNK signaling to EMT and aberrant motile capacity of MDR cells. By combinations of morphological, biological markers and functional analysis, we observed that KB/VCR cells possess mesenchymal properties with more potent motile capacity in comparison to KB cells, indicating that KB cells underwent EMT at the process of acquiring MDR. Furthermore, taking advantage of pharmacological tool and gene knockdown approaches, we demonstrated that JNK signaling may strictly be required for maintaining the mesenchymal properties of KB/VCR cells and its aberrant motile capacity through acting on snail and twist1, two critical transcriptional factors for EMT [10].

EMT, initially identified by its critical roles in developmental program of embryogenesis, has been demonstrated to be critical for numerous aspects of tumor progression, including proliferation and survival of tumor cells [19]. Moreover, transition of epithelial cancer cells to mesenchymal ones results in alterations in adhesive properties, activation of proteolysis and enhancement of motility, thereby endowing cancer cells the ability to invade and disseminate from primary location to distal organs sites and finally promoting metastasis of cancer [7,19]. Prior studies have shown that MDR cells acquire EMT transition compared to parental ones [20-22]. Aligned with these prior studies, our observations derived from KB/VCR and KB cells further validated this argument that cancer cells undergo EMT at the process of acquiring MDR. Thus, our data together with prior studies support that acquired mesenchymal properties may contribute, at least partially, to the aberrant motile and metastasis capacity of MDR cells arose during the process of chemotherapy.

JNK signaling has been implicated in numerous aspects of cancer progression, including the initiation, proliferation, survival and metastasis of cancers [11,23-26], as well as the occurrence of MDR during chemotherapy [11]. Emerging evidences have proven that JNK signaling promotes the metastasis of cancers, most likely through acting on matrix metalloproteinases [27-29], or on the small GTPases such as Rho A and Rac1 [29,30]. Taking advantage of a constitutively active JNK plasmid, a fusion protein of JNK and its upstream activator MKK7, Wang *et al.* recently reported that JNK activation can promote EMT in breast cancer cells [31]. In the current work, we observed that inhibition of JNK function via SP or knockdown of JNK expression may reverse the mesenchymal phenotypes of MDR cells KB/VCR. In addition, we further demonstrated that knockdown of c-Jun, a critical downstream transcriptional factor of JNK signaling, may disrupt the mesenchymal properties of KB/VCR cells as well. Many signaling pathways have been shown to be activated and play critical roles in maintaining the acquired chemoresistance, such as Notch and Hedgehog signaling pathways [32,33]. Hence, it is not surprising that Notch and Hedgehog signaling may be responsible for the activation of JNK signaling in KB/VCR acquired chemoresistant cancer cells. Indeed, we observed that JNK signaling was activated by the Hedgehog in acquired chemoresistant cancer cells via a cell autonomous manner, resulting in acquisition of EMT phenotype as presented in this study and activation of the Gli transcriptional factor of Hedgehog pathway (unpublished data from our lab). In turn, Gli activation may also increase the abundances of ABCB1 and ABCG2 [33,34]. Taken together, our data provide evidences that JNK signaling is strictly required for maintaining the mesenchymal properties of MDR cells, thus possibly involved in the aberrant metastasis capacity of MDR cells.

A variety of transcription factors including snail, slug, twist1, Zeb1 and Zeb2, to name a few, are involved in the EMT, through directly or indirectly regulating the expression of epithelial and mesenchymal markers, such as E-cadherin, N-cadherin *etc.*[19]. In this study, we observed that both snail and twist1 were elevated in the MDR cells KB/VCR in comparison to its parental KB cells, indicating the involvement of both snail and twist1 in the acquisition of mesenchymal phenotypes of MDR cells. This is discrepant to the finding from others that twist1 is solely involved in the EMT of drug resistant breast cancer cells acutely selected by lethal dose of adriamycin [20]. This discrepancy may be likely due to distinct systems used in these studies. We further found that the expression of snail and twist1 are both controlled by the JNK signaling, thus providing evidence that JNK signaling likely controls the mesenchymal phenotypes of MDR cells through acting on both snail and twist1. Our observations provide original interpretation of the molecular mechanisms for JNK signaling in regulation of EMT and in promotion of cancer metastasis.

Although great progresses have been made in the development and clinical usage of molecular target anti-cancer drugs, chemotherapy using conventional cytotoxic anti-cancer drugs is still one of efficient approaches for treatment of cancers, in despite of its limitations including MDR. Accumulating evidences have also shown that chemotherapeutic drugs usage can cause a secondary metastasis of survival cancer cells during chemotherapy and that MDR cancer cells possess aberrant metastatic capacity when compared to those sensitive to chemotherapeutic drugs [3-5]. In this regard, it is not surprising that the acquired more potent metastatic ability of MDR cells caused by traditional cytotoxic drugs usage may heavily hamper the chemotherapeutic efficacy, like MDR does. Furthermore, in addition to be involved in cancer metastasis, increasing evidences demonstrate that EMT program may also contribute to the occurrence of MDR through regulating the properties of cancer stem cell, which is significantly resistant to chemotherapy drugs [35-37]. MAPKs, such as ERK, JNK, and p38MAPK, are activated in multiple drug resistance cells [38]. However, the role of JNK activation in acquired chemoresistance still remains controversial [39]. Hence, this study may provide indications for interpreting the contributions of JNK signaling to MDR, through controlling the mesenchymal properties of MDR cells via acting on snail and twist1. Indeed, we observed that interfering with the expression of snail or twist1, which were both controlled by the JNK signaling as observed in this study, led to circumvent the MDR of KB/VCR cells (data to be published).

## Conclusions

In conclusion, the finding that JNK signaling may control mesenchymal properties of MDR cells KB/VCR via snail and twist1 implicates a potential therapeutic target for improving limitations and efficacy of chemotherapy, ranging from reversal of MDR to prevention of secondly metastasis caused by chemotherapeutic drugs usage.

## Competing interests

The authors declare that they have no competing interests.

## Authors’ contributions

XZ and XF conducted the experiments and are involved in data analysis. YK contributed to PCR assay and western blot analysis. XZ helped with drafting the manuscript. YC and WT designed the study, analyzed, and interpreted data, and drafted the manuscript. All authors read and approved the final manuscript.

## Pre-publication history

The pre-publication history for this paper can be accessed here:

http://www.biomedcentral.com/1471-2407/13/180/prepub
